# Microscale Cohesive-Friction-Based Finite Element Model for the Crack Opening Mechanism of Hooked-End Steel Fiber-Reinforced Concrete

**DOI:** 10.3390/ma14030669

**Published:** 2021-02-01

**Authors:** Yassir M. Abbas

**Affiliations:** Civil Engineering Department, King Saud University, Riyadh, P. O. Box 800, Riyadh 11421, Saudi Arabia; yabbas@ksu.edu.sa

**Keywords:** SFRC, finite element method (FEM), hooked-end fiber, micro-structure, fiber–matrix interface, cracking

## Abstract

The entire mechanical properties of steel fiber-reinforced concrete (SFRC) are significantly dependent on the fiber–matrix interactions. In the current study, a finite element (FE) model was developed to simulate the pullout response of hooked-end SFRC employing cohesive–frictional interactions. Plain stress elements were adapted in the model to exemplify the fiber process constituents, taking into consideration the material nonlinearity of the hooked-end fiber. Additionally, a surface-to-surface contact model was used to simulate the fiber’s behavior in the pullout mechanism. The model was calibrated against experimental observations, and a modification factor model was proposed to account for the 3D phenomenalistic behavior of the pullout behavior. Realistic predictions were obtained by using this factor to predict the entire pullout-slip curves and independent results for the peak pullout load. The numerical results indicated that the increased fiber diameter would alter the mode of crack opening from fiber–matrix damage to that combined with matrix spalling, which can neutralize the sensitivity of the entire pullout response of hooked-end steel fiber to embedment depth. Additionally, the fiber–matrix bond was enhanced by increasing the fiber’s surface area, sensibly leading to a higher pullout peak load and toughness. The developed FE model was also proficient in predicting microstructural stress distribution and deformations during the crack opening of SFRC. This model could be extended to fully model a loaded SFRC composite material by the inclusion of various randomly oriented dosages of fibers in the concrete block.

## 1. Introduction

### 1.1. Background

Steel fiber-reinforced concrete (SFRC) is a material created by incorporating arbitrarily distributed discontinuous steel fiber into a concrete matrix. This composite material is more ductile and has lower production cost (as it can be produced with less labor power) than conventional reinforced concrete. The steel fibers favorably enhance the cracked concrete response by controlling the crack propagation through the fiber’s toughening actions [1,2,3]; however, under biaxial loading, this advantage of fiber crack bridging unexpectedly vanishes [4]. Fiber toughness can be evaluated by using the pullout load versus crack opening response. The SFRC behavior under tension relies on the properties of the fiber (content, orientation, geometry, and material), cementitious matrix, and fiber–matrix interface [5]. However, the latter phase is generally considered as the “soul” of the SFRC, as it facilitates arresting the stressed crack tips (i.e., it is likely to alter the brittleness to a ductile response) [6,7].

The use of deformed fibers in SRFC is quite common. These fibers display superior ductility (throughout the pullout actions) compared to undeformed ones. Given the fiber’s synthetic deformation and the matrix’s strength, the bond capacity and pullout toughness of the deformed fibers are 3–7 times higher than those for straight fibers [8]. Hooked-end steel fibers are the most broadly recruited deformed fibers [9]. A hooked-end fiber with 20 mm depth in the concrete showed a maximum pullout load of ~350% more than the equivalent straight fiber [8]. Moreover, the hooked-end fiber’s energy dissipation during its pullout is superior to the smooth/straight fibers’ due to the accompanying plastic deformations [5]. It is worth noting that limited analytical models for predicting the pullout response of deformed fibers are available in the literature (e.g., [8,9]), which may be attributed to the extremely complex fiber–matrix interactions [7]. Moreover, the improved deformability of the SFRC is classically measured by the adjustment of the intrinsic strain-softening law of plain concrete [10]. A friction-based pulley simulation with two inelastic hooks to model the pullout response of hooked-end fiber was proposed by Alwan et al. [11]. Based on this simulation method, a model for the pullout behavior of multiple-hooked-end fibers (4D and 5D) was developed [12]. 

### 1.2. Significance of the Study

**Figure 1:**
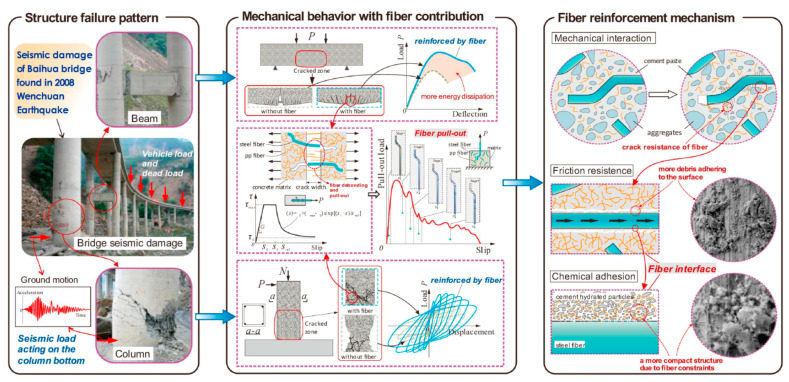
The significance of fiber inclusion in enhancing the mechanical performance of concrete at different scales [22]. Reprinted with permission from published by “Elsevier”, [2021].

As conceptually demonstrated in Figure 1, the fiber–matrix interactions govern the entire performance of a SFRC structure at various scales [13,14,15]. A cracked fiber-reinforced material can tolerate tensile stresses due to the bond of the fiber–matrix interface [16]. The pullout mechanism of a fiber is associated with debonding or rupture failure modes [17]. The fracture of loaded fibers is detected if the shear strength of the matrix is more than the tensile strength of the fiber. Inadequate material ductility was observed under the fiber’s rupture failure mode conditions, which resulted in underemployment of the fiber reinforcement capacity. In contrast, a composite material with super energy dissipation capability is obtainable if the tensile strengths of the fiber and matrix are comparable [18]. The classical way to quantify the influence of the fiber’s inclusion in a cement-based material is by acquiring the fiber’s bond-slip curve. This curve provides in-depth insight into the fiber–matrix interactions, and therefore aids additional enhancement of the composite material [19]. Research evidence has shown that the development a SFRC with super strength and ductility characteristics could be possible on the condition that the tensile strength of the fiber–matrix transition zone was comparable to that for matrix [20]. Additionally, the fiber’s interfacial area and orientation with the applied tensile loading are of great significance in the capacity of the fibers to control the cracking of the composite material [21]. 

The straightforward approach for designing an SFRC is through experimental investigations; however, it is both cost-prohibitive and complicated. Therefore, the performance-based optimization of the SFRC can be significantly aided by the use of numerical techniques. The finite element method (FEM) is a weighted residual-based numerical technique used to solve partial differential equations. This method is widely employed in engineering fields, as its implementation does not need a deep understanding of mathematics [23,24]. Recently, FEMs have been successfully used to characterize the mechanical features of the hooked-end SFRC [15,25]. Additionally, FEM has been used to model matrix fracture using individual fibers’ pullout response by segmenting the entire body [26]. 

### 1.3. Research Gap and Objectives

Fiber–matrix connectivity has been effectively modeled by nonlinear springs, in which the 2D simulations display improved modeling capabilities compared to the 3D versions [27]. The complexity emerging in developing a 3D numerical model for deformed fibers, and the relevant solution’s approximations notably curtail its precision for predicting the pullout force-slip curve. In contrast, 2D simulations can result in superior modeling of this curve, indicating the practicality of 2D modeling. Moreover, the analysis cost of 2D simulations is notably less than those developed with 3D models [28]. The analysis period of an individual hooked-end fiber pullout by a well-refined 3D-FE model using a standard CPU must run for about 20 h [25].

The innovative use of the mixed-mode cohesive fiber–matrix interfacial surface model has reliably been used to simulate the full behavior of inclined, undeformed fiber [20,21]. This model postulates that fiber–matrix interactions have three ingredients: interior physio-chemical bonding, surface friction, and normal pressure. According to this surface-based model, fiber bridging actions in a fiber-reinforced cementitious composite (FRCC) were investigated using FEM [29]. In this model, the fiber–matrix interface was considered to have inferior strength and stiffness. Moreover, the yield surface criterion and Coulomb friction for debonding and the subsequent stage were employed. Additionally, fiber crossing a crack of the matrix was simulated by high-order springs. Using surface-based interaction modeling, the FE model can be controlled to embody the full pullout response (i.e., adhesive bond, debonding, and frictional interactions) of the hooked-end fiber [1]. 

Even though many simulation schemes have been established, the existing research has many expediencies, reliabilities, and uninterrupted simulation issues for the microstructural fracture mechanics of SFRC (i.e., single fiber pullout). Simulation disturbance is commonly caused by numerical discrepancies and is associated with the nonlinearity of the fiber–matrix bond [30]. Therefore, stable simulation of the single hooked-end fiber pullout model is scarce. This dilemma is propagated further by the numerous influential factors of the fiber–matrix fracture [2]. The FEM parameters (e.g., mesh sensitivity, element type, and modeling of the interactions) increase the problem’s complexity. Despite decades of research, this continues to be highly debated, with inconsistent conclusions among researchers, and an insightful understanding of the biaxial stresses of SRFC has not yet been fully established. Consequently, this investigation’s objective was to develop an undisturbed, streamlined, and reliable FE model to simulate the fracture mechanism of the hooked-end SFRC at the microscale level. 

## 2. Pullout Response of Hooked-End Fibers

**Figure 2:**
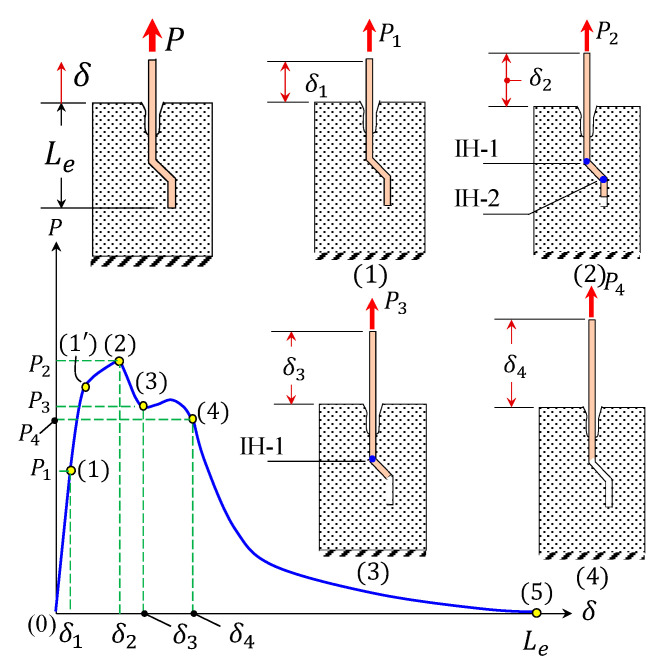
Pullout process for a hooked-end steel fiber [7,30].

Figure 2 displays the characteristic pullout response of a hooked-end fiber in the course of a concrete crack opening mechanism. This response can be broken into three phases: pure elastic (complete bonded), debonded, and frictional slipping. Table 1 presents an explanation of the stages of the pullout-slip curve of a hooked-end steel fiber.

## 3. Finite Element Modeling 

### 3.1. Geometry and Bond-Slip Model

In the current study, a general-purpose FE program (ABAQUS^®^, Johnston, RI, USA) was employed to handle the single fiber pullout problem. The bond-slip curve for various configurations was established by a Newton-based solution of the nonlinear (due to the considerable fiber’s slip effects) static equation of this problem. In this analysis, the steady-state fiber–matrix relative slip was modeled by applying a constant displacement velocity to the fiber’s upper tip. It is worth noting that a predefined traction–separation response could be employed instead of the bond-slip curve as a modification of the Bruggeling et al. [32] model for the pullout behavior of a steel bar. 

**Figure 3:**
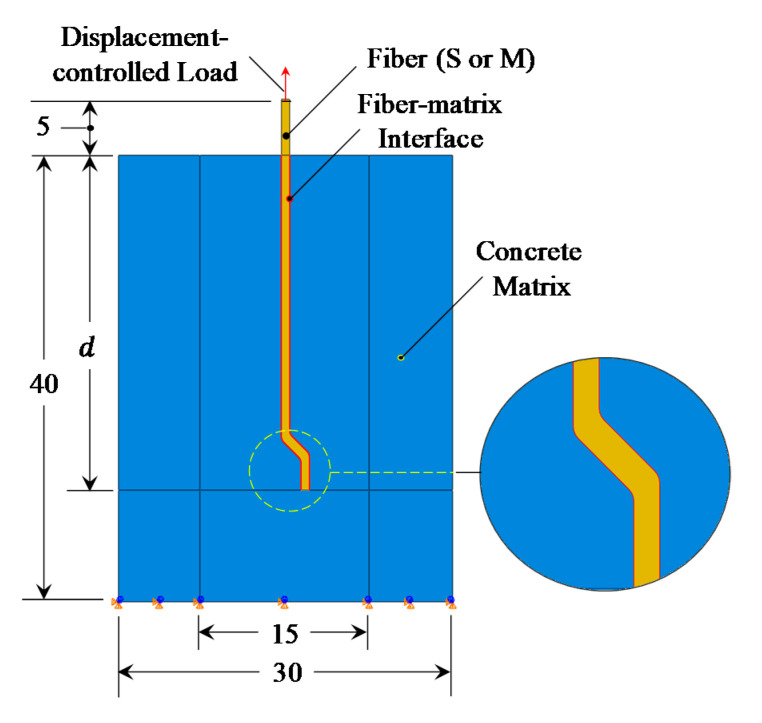
Finite element model of the fiber pullout process (all dimensions are in mm).

**Figure 4:**
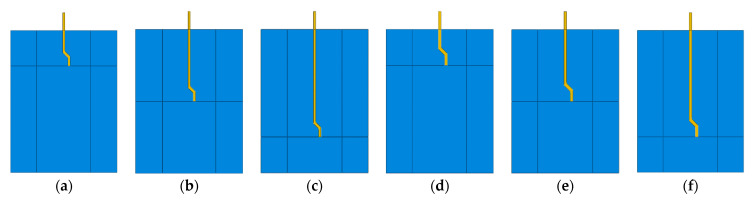
The modeling matrix: (**a**) S-10, (**b**) S-20, (**c**) S-30, (**d**) M-10, (**e**) M-20, and (**f**) M-30.

As shown in Figure 3, the fiber pullout process modeling involves the simulation of the following parts/interactions: the concrete matrix, the fiber, and the fiber–matrix interface. The fiber–matrix interactions are comparable to those for a steel bar embedded in concrete [1]. Several systems for simulating the bond-slip of steel rebar in concrete have been suggested in the literature [30,31,33]. Here, the surface-to-surface contact model [34] was used to simulate the fiber–matrix bond-slip response, which requires selecting the master and slave surfaces. This standard contact simulation was chosen, as it is capable of modeling all of the bond-slip interfacial mechanisms of the fiber pullout (i.e., adhesive bonding, debonding, and friction). The use of the “adhesive-friction-slip” model (i.e., surface-based adhesive response) offers a smart technique for simulating the cohesive interactions of insignificantly tiny interface widths. In the current investigation, it is worth noting that the fiber edges were selected as the master surface due to their high rigidity compared to the interfacial surface (Figure 3). In the current investigation, six FE models (Figure 4) were developed with two different fibers (i.e., S- and M-fiber, Table 2).

### 3.2. Fiber–Matrix Interactions

#### 3.2.1. Traction–Separation Response

In the current investigation, the linear biaxial uncoupled elastic traction–separation interaction (cohesive surface interactions) was assumed to model the fiber–matrix adhesive bond. This elastic response is expressed in Equation (1) as a linear constitutive formula relating the traction stress tensor (t) and corresponding slip displacements (δ) by the elastic stiffness matrix (K). This elastic model was defined for the two effective directions (n is the normal and s is shear axes).

(1) $$t=\left\{\begin{array}{c} t_{n}\\ t_{s} \end{array}\right\}=\left[\begin{array}{cc} k_{nn} & 0\\ 0 & k_{ss} \end{array}\right]\left\{\begin{array}{c} \delta_{n}\\ \delta_{s} \end{array}\right\}=K\delta$$

**Figure 5:**
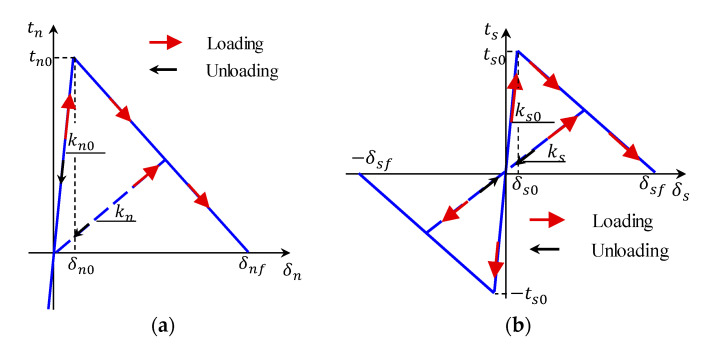
The traction–separation rule: (**a**) normal direction and (**b**) tangential direction [36].

**Figure 6:**
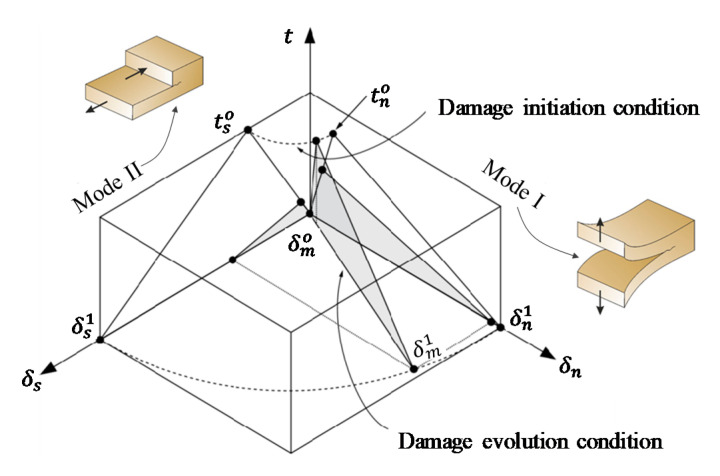
Mixed failure modes of the traction–separation rule [34].

Like the $t_{n}$-$\;\delta_{n}$ relationship (Equation (1)), the traction–separation rule can be used to model the fiber’s debonding process [35]. This rule (Figure 5) correlates the interfacial stress (traction) and corresponding split-up relative displacement (slip) of two surfaces. It is worth noting that the area below the traction-slip lines constitutes the energy dissipated in the material for complete failure (i.e., fracture energy). Generally, two failure modes (i.e., opening and sliding; Figure 6) govern the traction–separation rule. These modes (I and II) define the failure mechanism in the perpendicular and the two shearing directions, respectively. A combined fracture mode (Figure 6) likely ensues throughout the simulation process; however, the two fracture patterns could be autonomously defined [36,37,38]. 

#### 3.2.2. Damage Initiation and Evolution

On the one hand, the definition of the commencement of the fiber’s deprivation of the cohesive behavior at the maximum stressed contacted node is known as the “damage initiation” criterion. This condition is considered to exist when its value is equal or more than unity. In this investigation, the quadratic separation criterion [Equation (2)] was employed to model the damage initiation. In this formula, $\delta_{n}^{o}$ and $\delta_{s}^{o}$ denote the highest contact relative displacement, once it is along the normal or shear planes, respectively. In Equation (2), it is worth noting that Macaulay bracket (i.e., ⟨ ⟩) is used to indicate that the compressive interaction does not involve damage initiation.

(2) $${\left\{\frac{\left\langle \delta_{n}\right\rangle }{\delta_{n}^{o}}\right\}}^{2}+{\left\{\frac{\delta_{s}}{\delta_{s}^{o}}\right\}}^{2}=1$$

On the other hand, the damage evolution criterion expresses the rate of degradation of the cohesive stiffness after initiation. The damage evolution in this study was postulated to be based on the controlling displacement. Moreover, the exponential softening rule of the damage [Equation (3)], which describes the initiation to complete failure evolution, was employed in terms of the damage parameter, D, which varies between 0 (no failure) and 1 (complete failure). In this rule, $\delta_{m}^{o}$, $\delta_{m}^{f}$, and $\delta_{m}^{\max}$ stand for the slip at the damage initiation, complete failure, and maximum displacement obtained throughout the pullout process, respectively. Additionally, the parameter α is a scalar quantity expressing the material’s speed of damage evolution. 

Once D had been calculated, the resultant biaxial stresses were evaluated using Equation (4), in which $\bar{t}$ stands for the pre-fracture (elastic) stress. Additionally, Equation (5) was employed to calculate the resultant slip ($\delta_{m}$) corresponding to damage evolution with a combination of failure modes. Moreover, the degree of mode combinations (∅ in Equation (6)) was measured by the comparative magnitudes of normal and shear stresses. It is worth noting that ∅ ranges between 0 (pure tensile damage) and 1 (pure shear damage). Additionally, the “node-to-surface” connections with finite sliding were employed to model the fiber-to-matrix interactions. This discretization method was associated with the “slave” to “master” penetration issue, which was controlled by applying an initial node-to-node mesh for the two attached elements, appropriate element type, and mesh refinement for the interfacial zone.

(3) $$D=1-\left\{\frac{\delta_{m}^{o}}{\delta_{m}^{\max}}\right\}\left\{1-\frac{1-\exp\left(-\alpha \left(\frac{\delta_{m}^{\max}-\delta_{m}^{o}}{\delta_{m}^{f}-\delta_{m}^{o}}\right)\right)}{1-\exp\left(-\alpha \right)}\right\}$$

(4) $$\left\{\begin{array}{c} t_{n}\\ t_{s} \end{array}\right\}=\left(1-D\right)\left\{\begin{array}{c} {\bar{t}}_{n}\\ {\bar{t}}_{s} \end{array}\right\}$$

(5) $$\delta_{m}=\sqrt{\langle \delta_{n}\rangle^{2}+\delta_{s}^{2}}$$

(6) $$\emptyset =\frac{2}{\pi }\tan^{-1}\left(\frac{t_{s}}{t_{n}}\right)$$

In this investigation, the fiber-to-matrix’s tangential frictional response was modeled using Amontons’ third law of friction. Given that law, the maximum frictional resistance force [Equation (7)] exerted during the fiber pullout process in the fiber–matrix interface was evaluated in terms of normal bonding load ($N_{b}$) and the associated coefficient of dry friction (μ). It is worth noting that the intact edges of the fiber-to-matrix interface were those having a tangential force less than $\hat{F}$. Additionally, ABAQUS’s penalty-based approach was adapted to model the fiber–matrix tangential behavior, as it allows the relative slippage of the two attached surfaces with a low computational cost. Moreover, the “hard” contact pressure-overclosure (also penalty-based) formula was employed to simulate the fiber–matrix interface interactions in the perpendicular direction. This formula enabled the evaluation of $N_{b}$ based on the condition of intact edges. The numerical model employed coupled debonding and friction with exponential softening over the fiber–matrix interface. Initially, the friction response was regarded as not engaged, whereas the debonding was entirely governing (i.e., elastic response controlled by the adhesive fiber–matrix bonding). The frictional interactions were added to the normal ones as the damage initiation criterion had been fulfilled, whereas frictional contact entirely ruled the pullout loading if full debonding ensued. The employed parameters for the fiber–matrix interactions are given in Table 3.

(7) $$\hat{F}=\mu N_{b}$$

### 3.3. Material Models

In the current FE model, the steel and concrete materials were created using the property module and assigned to their sections in ABAQUS^®^. The primary material properties used in the development of the FE model will be presented in Section 4.1.

The steel was modeled using the “elastic” and the “classical metal plasticity” model, which employs the von Mises yield criterion available in the mechanical materials library. This material model enabled perfect plasticity with isotropic hardening (i.e., yield and plastic flows), which is suitable for monotonic excitations with no creep significance [34]. The plastic behavior of steel was established by the evaluation of true stress ($f_{true}$), and the corresponding inelastic strains ($\varepsilon_{true}^{inel}$) using Equations (8) and (9), respectively.

(8) $$f_{true}=f_{obs}\left(1+\varepsilon_{obs}\right)$$

(9) $$\varepsilon_{true}^{inel}=ln\left(1+\varepsilon_{obs}\right)-f_{true}/E_{s}$$

where $f_{obs}$ and $\varepsilon_{obs}$ are the observed stress–strain curves of steel under monotonic and quasi-static tensile loading, and $E_{s}$ is the modulus of elasticity for the steel. 

The concrete material was assumed as a perfectly elastic material. This simplified material model was proposed, as it had been concluded by previous investigators [1,8] that modeling the concrete in the 2D model with the damage plasticity model had resulted in unrealistic matrix element removal. 

### 3.4. Mesh Sensitivity and Properties

**Figure 7:**
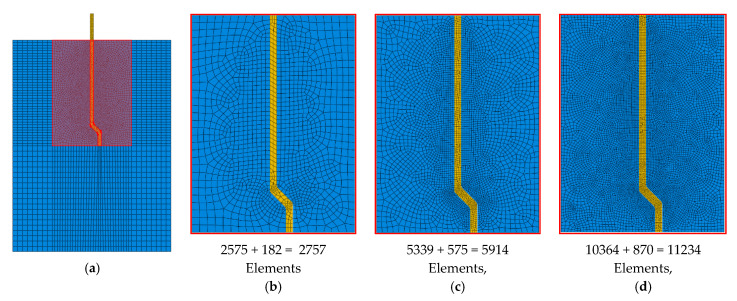
(**a**) Meshing the fiber (M-30)-matrix zone, (**b**) coarse-, (**c**) medium-, and (**d**) fine-discretization systems.

**Figure 8:**
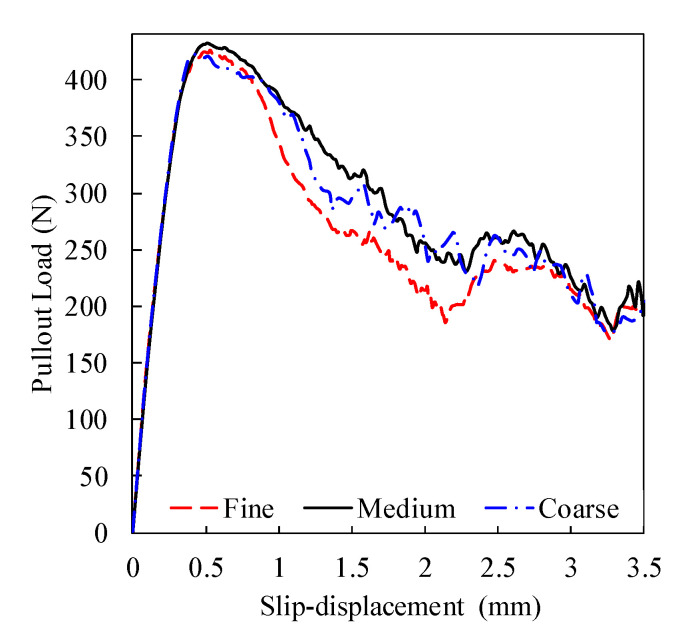
Load displacement curves for fine, medium, and coarse mesh systems.

**Figure 9:**
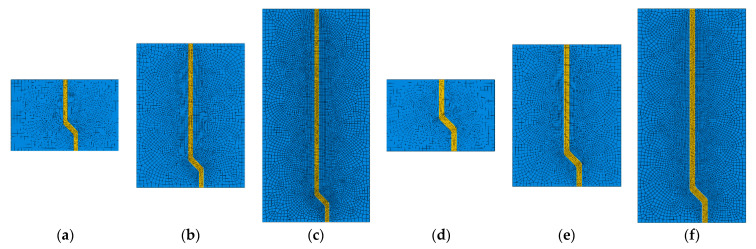
The meshing systems of the various fiber–matrix zones for multiple models (Figure 3) (**a**) S-10, (**b**) S-20, (**c**) S-30, (**d**) M-10, (**e**) M-20, (**f**) M-30.

Mesh optimization is a necessary step that balances the precision and the cost of the analysis. In Figure 7, three (i.e., coarse, medium, and fines) discretization systems were made for the fiber–matrix zone (highlighted in Figure 7a) of the M-30 model (with 2757: 5914 and 11,234 elements, respectively). Figure 8 presents the pullout load versus slip displacements for these discretization systems. This curve was fairly identical for the three mesh refinements at the elastic and incomplete debonded stages (Figure 9 and Table 1), with insignificant differences in the peak pullout load. Additionally, the coarse mesh showed an unsteady post-peak load response (which could be attributed to the unstable crack growth at this mesh size) that was markedly incompatible with the characteristic pullout response of a hooked-end fiber (Figure 2). As the medium mesh yielded a load-slip behavior comparable to that of the fine mesh and less simulation time, it was selected for the analysis of all developed models.

Using a medium-size mesh, Table 4 and Figure 9 show the mesh systems of the fiber–matrix zone (highlighted in Figure 7a) for the developed FE models, where a gradient mesh system was adapted to enhance the simulation performance. The element’s sizes were 1000, 200, and 50 µm at the model’s border, and the straight and curved parts of the fiber, respectively. As described in Section 3.2.2, a node-to-node connection was initially made for the fiber and the matrix to facilitate the definition of the relevant mixed-mode interfacial transition zone. Additionally, the CPS8R (eight-node reduced integration plane stress element) element type was assigned to the fiber and concrete parts. To facilitate the reproduction of these models, the associated flow chart and the keywords for “M-10” model are presented in Appendix A and Appendix B, respectively.

## 4. Calibration of the FE Model 

In this study, a fiber pullout test was experimentally performed on various concrete specimen configurations to investigate the validity of the established FE model. The results of these experiments were employed for comparison purposes with their comparable numerical ones. Accordingly, 18 typical concrete samples were tested under uniaxial fiber pullout and standard curing conditions (three replicated specimens for S-/M-10, S-/M-20, and S-/M-30) at the age of 90 days. 

### 4.1. Materials

In this experimental program, two types of hooked-end fibers were used (S and M, Table 2), and embedded in concrete at 10-, 20-, and 30-mm depths. The properties of the recruited hooked-end steel fibers are presented in Table 5. Additionally, type I Portland cement (satisfying the ASTM C150 specifications) was employed to develop the concrete mixture. The quantities of constituent materials of concrete mixtures are given in Table 6. The maximum aggregate size of these mixtures was 10 mm. It should be mentioned that a polycarboxylate-based superplasticizer (with a dosage of 1.1 L/m^3^) was incorporated in the mixtures to control the workability (with a slump in the range of 60–80 mm). The 28-day tensile and compressive strengths of the concrete (according to ASTM C496 and ASTM C39) were 2.6 MPa and 42.6 MPa, respectively. The elasticity modulus and Poisson’s ratio (as per ASTM C469) for the concrete were 30.5 GPa and 0.19, respectively. 

### 4.2. Method of Specimen Preparation and Testing

**Figure 10:**
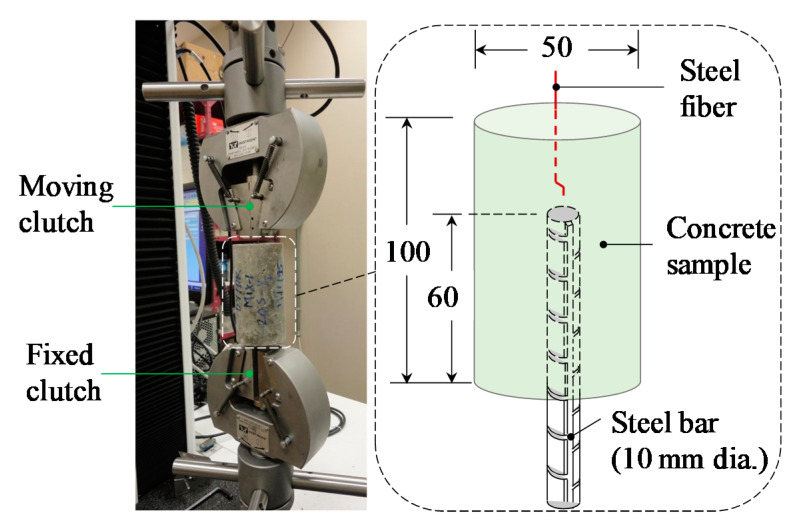
Fiber pullout test specimen and setup (all dimensions are in mm).

In the current investigation, cylindrical [50 mm (dia.) × 100 mm (ht.)] concrete specimens were used to perform the fiber pullout test (Figure 10). The concrete samples were released from the molds after casting for one day and reserved in a standard curing water tank until the age of testing (90 days). A universal testing machine (with 30 kN loading capacity) was used to conduct the fiber pullout test. This test setup involved gripping the steel bar of the concrete specimen in the machine’s fixed clutch and holding the steel fiber in the moving one (Figure 10). The loading of the fiber pullout test was performed under displacement-controlled conditions at a rate of 1.5 mm/min. It is worth noting that the toughness associated with fiber pullout was assessed by calculating the area under the pullout load-slip curve in the range of 0–4 mm slip displacements. It should be noted that the detailed information on the experimental methods is available in [39]. 

## 5. Results and Discussion 

### 5.1. Load-Slip Curves

**Figure 11:**
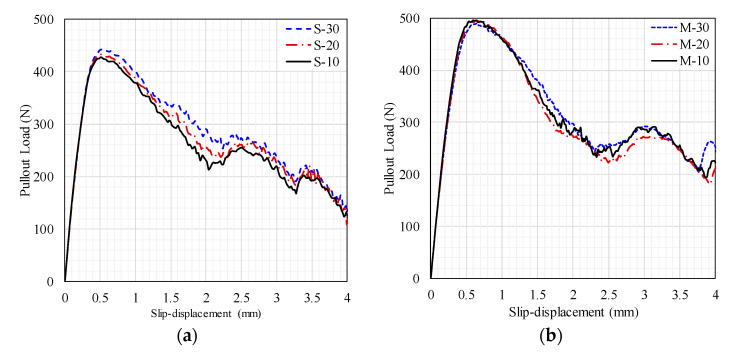
FE model results for the pullout load-slip responses: (**a**) S-fiber and (**b**) M-fiber.

Figure 11 shows the pullout load-slip curves produced by the FE models. As expected, the pullout ultimate load and toughness of the M-fibers were generally higher than those of the S-fibers, which could be attributed to the increased fiber–matrix bond resulting from the increased fibrous surface area. Additionally, Figure 11 shows a sensible result concerning the increased pullout maximum pullout load and toughness, with increased fiber depth for the 0.625 mm diameter fiber. However, a similar observation was not obtained for the M-fiber series (Figure 11b). In other words, no significant variations in the pullout load-slip response were observed for varying fiber depths. This pullout behavior could be attributed to the combined fiber–matrix failure and concrete spalling for fibers with shorter embedment depth and higher diameter. This result is in agreement with that reported by Deng et al. [22] for the pullout response of a hooked-end steel fiber embedded in hybrid FRCC.

**Figure 12:**
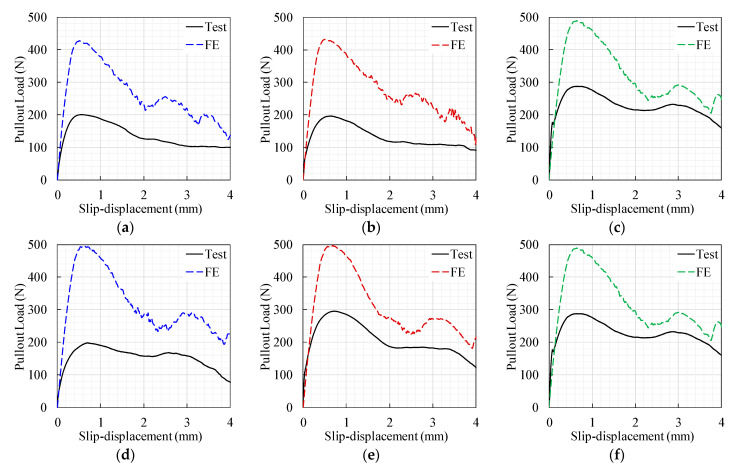
Model versus experimental load-slip curve results. (**a**) S-10, (**b**) S-20, (**c**) S-30, (**d**) M-10, (**e**) M-20, and (**f**) M-30.

The comparison between the FE model results and the mean of the observed experimental results is given in Figure 12. This figure depicts that both findings exhibited similar behavior; however, significant variations in magnitude existed. A notable overestimation of the FE model could be attributed to the fiber’s intrinsic 3D actual pullout actions, which are difficult to predict using 2D simulations. Overall, these findings are consistent with those reported by Van der Aa [1], Needleman [35], and Breitenbücher et al. [25]. 

**Figure 13:**
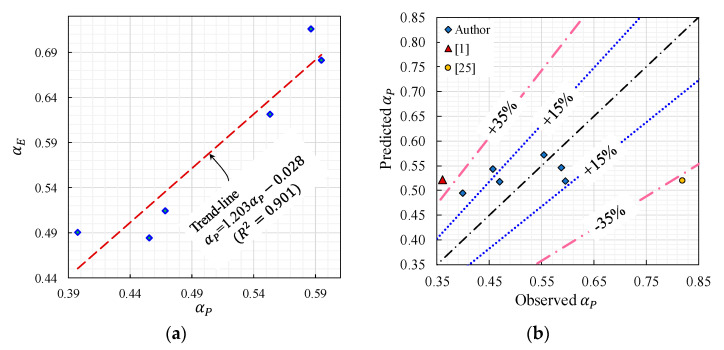
(**a**) Relationship between $\alpha_{E}$ and $\alpha_{P}$; (**b**) predicted vs. observed $\alpha_{P}$.

Table 7 shows the maximum pullout load ($P^{\prime }$), slip ($\delta^{\prime }$) at $P^{\prime }$ and pullout toughness ($T^{\prime }$) obtained by FE and physical modeling. In Table 7, the ratios of the observed to FE model for $P^{\prime }$, $\delta^{\prime }$, and $T^{\prime }$ results (i.e., $\alpha_{P}$, $\alpha_{\delta }$, and $\alpha_{E})$ are also given. The values of $\alpha_{\delta }$ suggested that the FE model provided good predictions for $\delta^{\prime }$ with 1.17 and 0.15 values for the mean and standard deviation of $\alpha_{\delta }$, respectively. This finding suggested that no modifications were needed to adjust the slip-displacement results of the FE models. Moreover, Table 7 indicates that the ratios of $\alpha_{P}$ and $\alpha_{E}$ were linearly correlated (Figure 13a). For this reason, and to facilitate comparisons with previously reported results, the $\alpha_{P}$ was considered as the modification factor for the FE model results. 

A nonlinear regression model for the modification factor ($\alpha_{P}$) is presented in Equation (10). This regression equation can be used to adjust the pullout load data of the FE model. In the development of this equation, the fiber’s diameter (d) and embedment length ($L_{e}$) were employed as predictors. Equivalent data from the literature (Table 8) are included in the establishment of the prediction model of $\alpha_{P}$, to make the modification factor applicable to various hooked-end fibers embedded in normal concrete. The initial assessment of the correlations between the $\alpha_{P}$ and d yielded a polynomial trend, whereas the $\alpha_{P}$ and $L_{e}$ relationship exhibited exponential tendencies. It is worth noting that the parameters of the $\alpha_{P}$ prediction equation (i.e., A, B, and C) in Equation (10) were evaluated by the Gauss–Newton (with a tolerance of 1 × 10^−5^) and least-squares iterative procedures.

(10) $$\alpha_{P}=Ad^{-B}\left[\exp\left(CL_{e}\right)\right]$$

where A=0.438, B=0.25, and $C=4.996\times 10^{-3}.$

**Figure 14:**
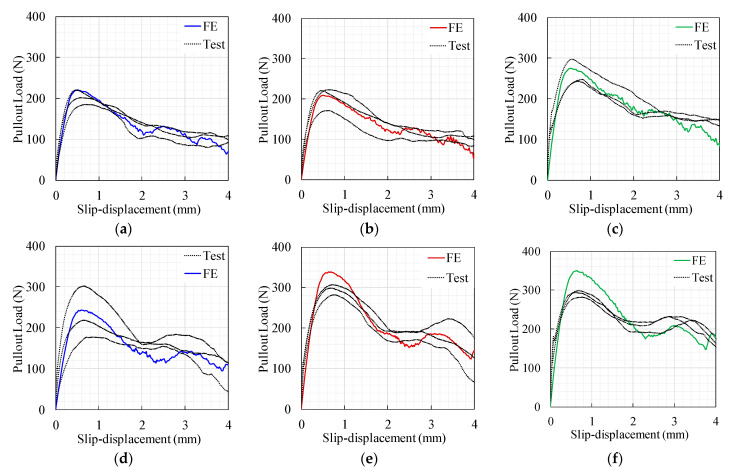
The modified model versus the experimental load-slip curve results. (**a**) S-10, (**b**) S-20, (**c**) S-30, (**d**) M-10, (**e**) M-20, and (**f**) M-30.

Table 8 shows that the average and standard deviation of the predicted to observed $\alpha_{P}$ were 1.058 and 0.073, respectively. This finding establishes that the model of $\alpha_{P}$ [Equation (10)] produced realistic predictions. The data of the observed–predicted modification factor are also plotted in Figure 13b. This figure displays the satisfactory prediction performance of Equation (10) for the FE model modification factor of the data of the current investigation, with an error band of about ±15%. However, a broader inaccuracy band (±35%) was observed for the independent data, which could be attributed to the variance in the physical and numerical modeling between these investigations and the current one. Therefore, the pullout load-slip modification factor proposed herein was limited to the reported testing conditions. The previously evaluated modification factor (Table 7) was employed to correct the pullout data of the un-modified FE model results (Figure 11 and Figure 12). The modified pullout curves are presented in Figure 14. This figure shows that the modified FE model results reasonably predicted the thorough pullout response of the different hooked-end fiber features. This post-modification processing involved the multiplication of the pullout load data (Figure 11 and Figure 12) of the FE model by $\alpha_{P}$ (Table 7). 

### 5.2. Fracture Pattern

**Figure 15:**
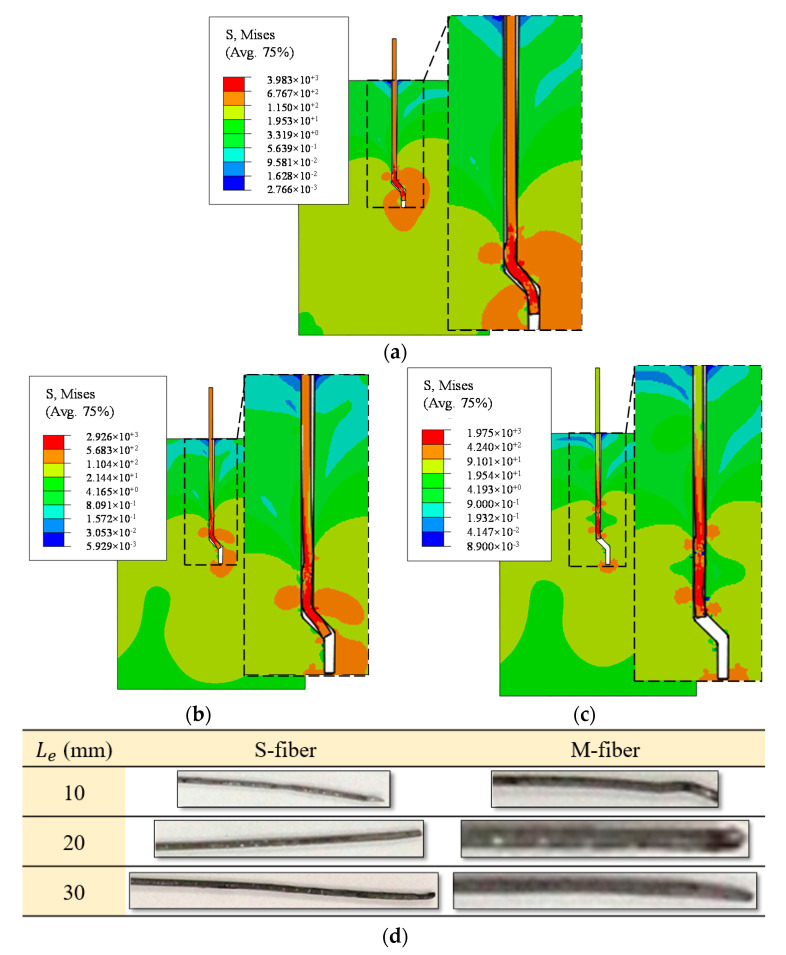
Stress contours during S20-fiber pullout. (**a**) δ= 1.634 mm, (**b**) δ= 3.244 mm, (**c**) δ= 5.076 mm, (**d**) fibers after the pullout test.

The von Mises stress distribution at different phases of the S20-fiber pullout process and the deformed fibers after the pullout test are presented in Figure 15. The simulated fiber-stress responses revealed that the fibers’ curved segments were ultimately the stressed regions during the fiber pullout. It is worth noting that Inglis and Kok [40] obtained similar results. Figure 15 also demonstrates that the stresses on these hooks decreased as the pullout progressed (i.e., stress relief occurred due to their plastic deformations). By comparing the shape of the fiber after the test (Figure 15d) and FE simulations (Figure 15a–c), it could be concluded that the developed FE model in the current study was also proficient in capturing the fiber and matrix deformations during the crack growth of SFRC.

## 6. Conclusions and Prospective Research 

In the current study, an FE model was used to simulate the pullout response of hooked-end SFRC employing cohesive–frictional mixed-mode interactions. Plain stress elements were adapted to model the constituents of the fiber’s pullout process, considering the material nonlinearity of the hooked-end fiber. Additionally, a surface-to-surface mixed-mode (i.e., cohesive-friction with various failure types) contact model was chosen to simulate the fiber’s complete response during the pullout process (including the nonlinear geometry interactions). The model was calibrated against experimental observations, and the modification factor model was proposed to account for the 3D phenomenalistic behavior simulated by 2D numerical modeling. This modification factor is likely limited to the numerical and physical modeling conditions of this study. Reasonable predictions were obtained by employing this factor to generate the entire pullout-slip curves of previous results, including the ultimate pullout load. Based on the numerical and experimental investigations conducted in this study, the following conclusions have been drawn:

- The FE models’ results confirmed that the increased area of the fiber’s surface was able to enhance the fiber–matrix bond, resulting in higher pullout ultimate load and toughness.
- Increasing the fiber’s diameter could alter the mechanism of crack opening from loss of the fiber–matrix bond to that combined with matrix spalling. The latter mode of failure can neutralize the sensitivity of the complete pullout response of hooked-end steel fiber to embedment depth.
- Plane stress elements could model the entire behavioral constituents of a hooked-end steel fiber’s pullout response from the concrete matrix. However, the simplification of the spatial stress-dependence during the fiber’s pullout by plane one is likely to result in pullout load overestimation.
- The benefits of 2D modeling of the fiber’s pullout were discussed using the pullout load scaling-down modification factor able to be predicted given the fiber’s size and depth into the matrix.
- In the present study, the developed FE model was also capable of capturing the stress distribution and deformations during the crack opening of a SFRC; therefore, it provided confirmatory coincidence.

The scope of the present investigation may be expanded in the future to include the following studies.

- Understanding the influence of concrete plasticity, shrinkage, and creep on deformed fibers’ pullout behavior.
- Insights into the effect of the matrix’s performance class (normal, high, and ultrahigh) on the fibers’ pullout behavior should be addressed. Moreover, deformed fibers’ pullout behavior under various confinement pressure and boundary conditions applied on the concrete surface has rarely been reported.
- A much broader understanding of the numerical solution scheme (i.e., implicit or explicit) on the FE model results’ stability and accuracy remains an open question.
- Simulation of the complete behavior of SFRC material under various loading conditions. This type of modeling could be performed by combining the influence of cracking and pullout of fibers. To approach this goal, a user subroutine needs to be written in ABAQUS^®^ for the inclusion of various randomly oriented dosages of fibers in the concrete block.

## Figures and Tables

**Table 1:** Phases of the pullout intrinsic response of a hooked-end fiber [7,30].

Phase	Sub-Phase	Range in Figure 2	Criterion	Description
Pure elastic	-	0–1	$\delta \leq \delta_{1}$	The hooked-end fiber acts as a straight one (deformed elastically) in this fully bonded phase, where the adhesive fiber–matrix governs the system.
Debonding	Incomplete debonded	1–2	$\delta_{1}<\delta \leq \delta_{2}$	Involves the fiber’s debonding process, and due to its deformability, this stage extended to (2), instead of (1′) in Figure 2, where the fiber’s lower end approaches the matrix’s first bend and plasticity begins. At the end of this phase, two inelastic hinges are formed (i.e., IH-1 and IH-2 in Figure 2).
	Disappearance of IH-2	2–3	$\delta_{2}<\delta \leq \delta_{3}$	The second inelastic hinge (IH-2) evanesces, as δ approaches $\delta_{3}$, which notably reduces the pullout load (due to the decrease in the mechanical and frictional bonding).
	Disappearance of IH-1	3–4	$\delta_{3}<\delta \leq \delta_{4}$	In this sub-phase, the fiber’s first inelastic hinge (IH-1) fades out. Debonding is completed as $\delta =\delta_{4}$. The slight load surge in this sub-phase is because the fiber travels through the second angle of the fiber–matrix interface.
Frictional slipping	-	4–5	$\delta >\delta_{4}$	The fiber becomes deformed-straight during this phase, where the Coulomb’s kinetic friction governs the fiber–matrix interactions. It is worth noting that a deformed fiber’s frictional abrasion is notably higher than that for a straight fiber [31].

**Table 2:** The reference points of the hooked-end steel fibers.

**Point**	**1**	**2**	**3**	**4**	**5**	**6**	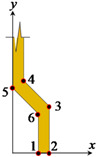
**S-fiber**							
x	1.473	2.098	2.098	0.625	0	1.473	
y	0	0	2.721	4.194	3.752	2.279	
**M-fiber**							
x	1.768	2.518	2.518	0.750	0	1.768	
y	0	0	3.265	5.033	4.503	2.735	

The radii of the fillets for the S- and M-fibers were 0.42 and 0.50 mm, respectively. All dimensions are in mm.

**Table 3:** Parameters of the cohesive properties of the fiber–matrix interface.

$k_{nn}$	$k_{ss}$	$t_{n}$	$t_{s}$	$\delta_{n}$	$\delta_{s}$	α	μ
(N/mm^3^)		(MPa)		(mm)			
11		0.7		0.5		6	0.05

**Table 4:** Mesh systems for the various fiber–matrix zones for the developed models.

Model	Number of Nodes			Number of Elements		
	Fiber	Matrix	Total	Fiber	Matrix	Total
S-10	1936	11,009	12,945	585	3560	4145
S-20	1971	16,525	8496	572	5360	5932
S-30	2620	22,388	25,008	755	7275	8030
M-10	1414	10,418	11,832	419	3365	3784
M-20	2208	16,262	18,470	647	5271	5918
M-30	3190	22,220	25,410	941	7215	8156

**Table 5:** Physicomechanical characteristics of the steel fibers.

Type	Diameter (mm)	Camber Length (mm)	Hook Length (mm)	Aspect Ratio	Hook’s Angle	Poisson’s Ratio	Elasticity Modulus (GPa)	Tensile Strength (MPa)
S-fiber	0.625	2.0	2.5	80	45°	0.3	210	1250
M-fiber	0.750	2.5	3.0					

**Table 6:** Constituent materials of the concrete (kg/m^3^).

Water	Cement	Aggregate	
		Coarse	Fine
157.5	350	1040	700

**Table 7:** FE and physical modeling of the fiber pullout parameters.

**Model**	$\mathrm{Max}\;\mathrm{Load}\;\left(P^{\prime }\right)\text{-}N$			$\mathrm{Slip}\;(\delta^{\prime }$$)\;\mathrm{at}\;P^{\prime }$ (mm)			$\mathrm{Toughness},\;T^{\prime }$ (×10^−3^ J)		
	$\mathrm{FE}\;(P_{FE}^{\prime })$	$\mathrm{Test}\;(P_{T}^{\prime })$	$\alpha_{P}=P_{T}^{\prime }/P_{FE}^{\prime }$	FE $\left(\delta_{FE}^{\prime }\right)$	$\mathrm{Test}\;(\delta_{T}^{\prime })$	$\alpha_{\delta }=\delta_{T}^{\prime }/\delta_{FE}^{\prime }$	$\mathrm{FE}\;(T_{FE}^{\prime })$	$\mathrm{Test}\;(T_{T}^{\prime })$	$\alpha_{E}=T_{T}^{\prime }/T_{FE}^{\prime }$
S-10	427.91	200.46	0.47	0.50	0.56	1.12	1062.94	547.08	0.51
S-20	432.47	197.14	0.46	0.51	0.64	1.26	1101.13	533.71	0.48
S-30	442.42	244.72	0.55	0.52	0.73	1.41	1149.70	714.27	0.62
M-10	495.53	197.16	0.40	0.60	0.70	1.18	1257.20	616.95	0.49
M-20	496.50	295.44	0.60	0.69	0.74	1.07	1223.43	833.34	0.68
M-30	489.58	287.15	0.59	0.65	0.65	1.00	1275.90	913.73	0.72
Average	464.07	237.01	0.51	0.58	0.67	1.17	1178.38	693.18	0.58
Std. ^1^	33.07	45.83	0.08	0.08	0.07	0.15	87.03	155.77	0.10
Min	427.91	197.14	0.40	0.50	0.56	1.00	1062.94	533.71	0.48
Max	496.50	295.44	0.60	0.69	0.74	1.41	1275.90	913.73	0.72
Range	68.58	98.31	0.20	0.18	0.17	0.41	212.96	380.02	0.23

^1^ Std. = Standard deviation.

**Table 8:** Observed versus predicted modification factor, $\alpha_{P}$.

	d (mm)	$L_{e}\;(\mathrm{mm})$	$\mathrm{Observed}\;\alpha_{P}\;(\alpha_{PO})$	$\mathrm{Predicted}\;\alpha_{P}\;(\alpha_{PP})$	$\alpha_{PO}/\alpha_{PP}$	$\mathrm{Std}.\;(\alpha_{PP},\;\alpha_{PO})$
Author	0.625	10	0.468	0.427	1.105	0.035
	0.625	20	0.456	0.488	1.194	0.063
	0.625	30	0.553	0.557	1.034	0.013
	0.750	10	0.398	0.460	1.243	0.068
	0.750	20	0.595	0.525	0.874	0.053
	0.750	30	0.587	0.600	0.932	0.028
[1]	0.900	30	0.360	0.457	1.450	0.115
[25]	0.750	20	0.820	0.820	0.634	0.212
-	-	-	Average		1.058	0.073

## Data Availability

Data is contained within the article.

## Appendix B. Keywords for “M10” Model

** PARTS

*Part, name=Matrix-1

*Element, type=CPS8R

*Surface, type=ELEMENT, name=Matrix-R

*Surface, type=ELEMENT, name=Matrix-L

** Section: Matrix

*Solid Section, elset=_PickedSet5, material=Concrete

*End Part

*Part, name=SF

*Element, type=CPS8R

*Surface, type=ELEMENT, name=SF_R

*Surface, type=ELEMENT, name=SF_L

** Section: Steel

*Solid Section, elset=_PickedSet6, material=Steel

*End Part

*Assembly, name=Assembly

*Instance, name=SF-1, part=SF

*End Instance

*Instance, name=Matrix-1-1, part=Matrix-1

*End Instance

*Nset, nset=RP

*Surface, type=ELEMENT, name=_PickedSurf20, internal

** Constraint: Constraint-2

*Coupling, constraint name=Constraint-2, ref node=_PickedSet21, surface=_PickedSurf20

*Kinematic

*End Assembly

** MATERIALS

*Material, name=Concrete

*Elastic, 33000., 0.2

*Material, name=Steel

*Elastic, 210000., 0.3

*Plastic

1000., 0.

1061., 0.02

1096., 0.05

1136., 0.1

1166., 0.15

1192., 0.2

1214., 0.25

1235., 0.3

1254., 0.35

1271., 0.4

1288., 0.45

1303., 0.5

** INTERACTION PROPERTIES

*Surface Interaction, name=Cohesive

*Friction, elastic slip=0.01 0.05,

*Cohesive Behavior, 11.,11.,11.

*Damage Initiation, criterion=MAXS, 0.7, 0.7, 0.7

*Damage Evolution, type=DISPLACEMENT, softening=EXPONENTIAL, mixed mode behavior=TABULAR, mode mix ratio=TRACTION, 0.5,6.,0.,0., 0.5,6.,1.,0.

*Damage Stabilization 0.

** BOUNDARY CONDITIONS

* Name: BC-1 Type: Displacement/Rotation

*Boundary

_PickedSet23, 1, 1

_PickedSet23, 2, 2

_PickedSet23, 6, 6

** INTERACTIONS

** Interaction: LL

*Contact Pair, interaction=Cohesive

SF-1.SF_L, Matrix-1-1.Matrix-L

** Interaction: RR

*Contact Pair, interaction=Cohesive

SF-1.SF_R, Matrix-1-1.Matrix-R

** STEP: Step-1

*Step, name=Step-1, nlgeom=YES, extrapolation=PARABOLIC, inc=1000000

*Static

0.0001, 1., 1e-30, 0.01

** BOUNDARY CONDITIONS**

** Name: BC-2 Type: Displacement/Rotation

*Boundary

RP, 2, 2, 4.

** OUTPUT REQUESTS

*Restart, write, frequency=0

** FIELD OUTPUT: F-Output-1

*Output, field, variable=PRESELECT

** HISTORY OUTPUT: H-Output-1

*Output, history

*Node Output, nset=RP

RF2, U2

*End Step
